# Trends in the Incidence of Ramsay Hunt Syndrome and Zoster Sine Herpete Following the Introduction of Routine Varicella Vaccination in Hokkaido, Japan

**DOI:** 10.7759/cureus.100403

**Published:** 2025-12-30

**Authors:** Yasushi Furuta, Hiroko Yanagi, Michiya Matsumura, Keishi Fujiwara

**Affiliations:** 1 Department of Otolaryngology-Head and Neck Surgery, Teine Keijinkai Hospital, Sapporo, JPN; 2 Department of Otolaryngology-Head and Neck Surgery, Faculty of Medicine and Graduate School of Medicine, Hokkaido University, Sapporo, JPN

**Keywords:** facial paralysis, ramsay hunt syndrome, varicella vaccination, varicella-zoster virus, zoster sine herpete

## Abstract

Background: Routine varicella vaccination was introduced in Japan in October 2014. A large-scale epidemiological study conducted in Miyazaki Prefecture subsequently reported a marked increase in the incidence of herpes zoster (HZ) among the child-rearing population aged 20-40 years following this introduction.

Objective: This study aimed to investigate long-term trends in the incidence of HZ-related facial palsy, Ramsay Hunt syndrome (RHS) and zoster sine herpete (ZSH), among patients with acute peripheral facial palsy (APFP) aged 15 years or older, treated at a single institution in Hokkaido, Japan, over a 15-year period (2007-2022), and to compare the proportions before and after the introduction of routine varicella vaccination.

Methods: This retrospective cohort study included 641 patients (15 years old or older) diagnosed with APFP, including Bell’s palsy, ZSH, and RHS, between July 2007 and December 2022 at a single institution in Hokkaido, Japan. Varicella-zoster virus (VZV) reactivation was diagnosed using an enzyme-linked immunosorbent assay with paired serum samples. The ratio of HZ was defined as the proportion of ZSH and RHS cases among all APFP patients.

Results: VZV reactivation was confirmed in 219 of 641 patients (34.2%). The proportion of HZ-related facial palsy was 33.6% (102/304) before the introduction of routine varicella vaccination and 34.7% (117/337) afterward, showing no significant overall change. However, among patients aged 15-29 years, the proportion of HZ significantly increased from 38.9% (14/36) before vaccination to 63.3% (19/30) after vaccination. This corresponds to an odds ratio of 2.714 (95% CI: 1.009-7.301), indicating that individuals in this age group had approximately 2.7 times higher odds of developing HZ-related facial palsy in the postvaccination period compared with the prevaccination period. The chi-square statistic (χ² = 3.910) and p-value (p = 0.048) further support that this increase is statistically significant.

Conclusions: Although the overall incidence of HZ-related facial palsy among APFP patients did not change significantly after the implementation of routine varicella vaccination in 2014, a marked increase was observed in younger patients aged 15-29 years. These findings suggest a need for heightened clinical awareness of VZV reactivation in young generation presenting with APFP in Hokkaido, Japan.

## Introduction

Varicella-zoster virus (VZV) is not eliminated from the body after primary varicella (chickenpox) infection. Instead, it remains latent in the sensory ganglia throughout life. Reactivation of latent VZV, typically occurring years or even decades later, results in herpes zoster (HZ, shingles). Ramsay Hunt syndrome (RHS), also known as herpes zoster oticus, is characterized by zoster lesions around the auricle or in the oropharynx, acute peripheral facial palsy (APFP), and symptoms involving the vestibulocochlear nerve. VZV reactivation can also cause APFP in the absence of skin lesions, a condition known as zoster sine herpete (ZSH). ZSH can be distinguished from Bell’s palsy using appropriate serological and/or molecular diagnostic methods [1].

RHS accounts for approximately 20% of APFP cases, and ZSH is diagnosed in 8-19% of patients initially presumed to have Bell’s palsy [2,3]. Consequently, VZV reactivation is the second most common cause of APFP, following Bell’s palsy. Clinically, APFP associated with VZV reactivation tends to be more severe and is associated with poorer recovery outcomes compared to Bell’s palsy [4].

In Japan, the varicella vaccine (Oka strain) had been available since 1987; however, until October 2014, it was administered only as a voluntary (nonroutine) vaccination. The uptake under this voluntary program remained relatively low, with reported national coverage of approximately 40% [5]. Consequently, prior to the introduction of routine childhood immunization in October 2014, the vaccine’s impact on overall varicella epidemiology was limited [5]. Routine varicella vaccination has led to a marked decline in annual varicella incidence [5,6].

Previous epidemiological studies analyzing HZ incidence by age group revealed a transient decline in individuals in their 30s, followed by a steady increase with advancing age [7]. This temporary reduction in incidence among people in their 30s has been attributed to the boosting effect, in which frequent exposure to pediatric varicella cases stimulates cell-mediated immunity against VZV in child-rearing adults [7,8]. With the widespread adoption of varicella vaccination and the resulting decline in natural varicella infections, it has been hypothesized that this boosting effect is diminished, potentially leading to a decline in VZV-specific immunity among adults and an increased risk of HZ reactivation [9,10]. A recent study reported an increased incidence of HZ following the introduction of routine varicella vaccination in Japan, particularly among the child-rearing population aged 20-40 years [11].

Therefore, to better understand the long-term epidemiological trends in HZ-related facial palsy (RHS and ZSH) in the context of routine childhood varicella vaccination, we conducted a retrospective study of patients aged 15 years or older with APFP treated at a single institution in Hokkaido, Japan. The aim of this study was to evaluate changes in the proportion of HZ-related facial palsy among these patients over a 15-year period (2007-2022) and to compare the proportions before and after the introduction of routine varicella vaccination.

## Materials and methods

Study population

This retrospective observational cohort study included patients aged 15 years or older who were diagnosed with APFP, including Bell’s palsy, ZSH, and RHS, and who presented to Teine Keijinkai Hospital between July 2007 and December 2022. All patients were treated within seven days of symptom onset.

Of the 767 eligible patients, 126 who did not undergo serological testing were excluded from the analysis. Consequently, a total of 641 patients with APFP were included in the final analysis. All patients were residents of Hokkaido Prefecture.

Study setting

Teine Keijinkai Hospital is a 660-bed tertiary care hospital located in Sapporo, Hokkaido, Japan, providing comprehensive emergency and outpatient services to a large regional population. The hospital serves as a major referral center for neurological and otolaryngological disorders, enabling consistent surveillance of APFP cases.

Data collection

Data were retrospectively collected from electronic medical records. Clinical data were extracted by the staff of the Medical Support Division. Extracted variables included patient demographics, clinical diagnosis (Bell’s palsy, ZSH, or RHS), symptom onset and timing of treatment initiation, serological test results, and residential area. Only patients with completed VZV serological testing were included in the final analysis.

Diagnosis of VZV reactivation in APFP

Paired serum samples were collected from all APFP patients at the initial visit and again 2-4 weeks later (convalescent phase). VZV-specific immunoglobulin (Ig) G and IgM antibodies were measured using an enzyme-linked immunosorbent assay (ELISA) performed at an external accredited laboratory (SRL Laboratory, Tokyo, Japan). According to the manufacturer’s criteria, VZV IgG titers <2.0 are interpreted as negative, and VZV IgM titers <0.80 are considered negative. VZV reactivation was diagnosed when either a ≥twofold increase in anti-VZV IgG titers was observed or when IgM antibodies were positive. In addition, patients with a high anti-VZV IgG titer (≥50 × 10² mIU/mL) were considered positive for VZV reactivation if an increase in anti-VZV IgG was confirmed in the convalescent sample [12,13].

RHS was diagnosed in patients with APFP who exhibited typical zoster lesions on the auricle, in the oropharyngeal epithelium, or who presented with cochleovestibular symptoms. All RHS cases had serologically confirmed VZV reactivation. ZSH was diagnosed in patients with no zoster lesions or cochleovestibular symptoms, but in whom serologic evidence of VZV reactivation was present. All remaining cases were diagnosed with Bell’s palsy.

Calculation of HZ rate

The annual rate of HZ-related facial palsy was calculated by dividing the number of patients with RHS and ZSH by the total number of APFP patients seen each year. The same method was applied to calculate the age-specific incidence rates.

Data analysis

Descriptive statistics were used to present the annual and age-stratified proportions of Bell’s palsy, ZSH, and RHS. The proportions of HZ-related facial palsy (ZSH and RHS combined) before and after the introduction of routine varicella vaccination were compared using chi-square tests. A two-sided p-value < 0.05 was considered statistically significant. We also calculated odds ratios with 95% confidence intervals to evaluate the effect size. All analyses were performed using EZR [14] (Saitama Medical Center, Jichi Medical University, Japan), which is a graphical user interface for R.

Ethical approval

This retrospective cohort study was conducted in accordance with the principles of the Declaration of Helsinki and approved by the Institutional Review Board of Teine Keijinkai Hospital, Sapporo, Japan.

## Results

VZV reactivation in APFP patients

Among the 641 patients diagnosed with APFP, 116 presented with typical zoster lesions on the skin or oral epithelium and were thus diagnosed with RHS. VZV reactivation in these patients was confirmed by ELISA. Of the remaining 525 patients tested using paired sera, 103 (19.6%) were diagnosed with ZSH based on evidence of VZV reactivation. Overall, 219 of the 641 APFP patients (34.2%) showed evidence of VZV reactivation at the onset of facial paralysis and were classified as having HZ-related facial palsy. The remaining 422 patients were diagnosed with Bell’s palsy.

Annual rate of HZ in APFP patients

**Figure 1:**
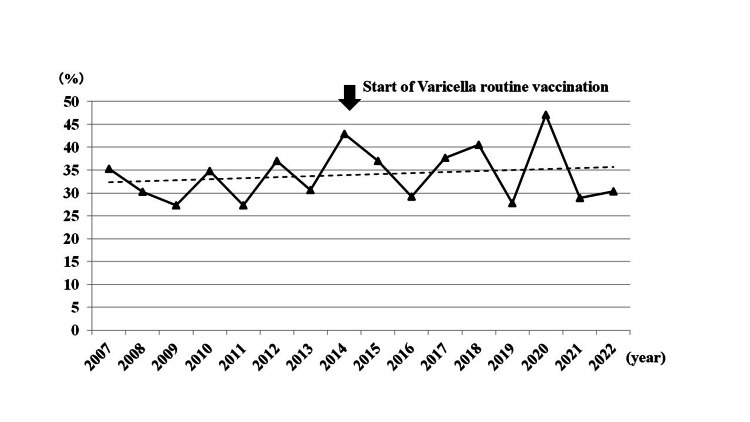
Annual trend in the proportion of herpes zoster-related facial palsy among patients with acute peripheral facial paralysis ZSH: zoster sine herpete; RHS: Ramsay Hunt syndrome The line graph depicts the annual proportion (%) of herpes zoster-related facial palsy (ZSH and RHS) among patients with acute peripheral facial paralysis from 2007 to 2022. The introduction of routine varicella vaccination (October 2014) is indicated by a vertical arrow

Table 1 summarizes the number of APFP patients per year. The annual proportion of HZ-related facial palsy (ZSH and RHS) during the 15-year study period is shown in Figure 1. The proportion was 33.6% (102/304 cases) before the introduction of routine varicella vaccination and 34.7% (117/337 cases) after its implementation (Table 2). There was no significant difference between the two periods (odds ratio, 1.053; 95% CI, 0.760-1.460; χ² = 0.097; p = 0.803).

**Table 1:** Annual number of patients with acute peripheral facial paralysis in each year APFP: acute peripheral facial palsy; ZSH: zoster sine herpete; RHS: Ramsay Hunt syndrome Annual number of patients with APFP, including Bell’s palsy, ZSH, and RHS between 2007 and 2022. The table shows the yearly case counts used to calculate the annual proportion of herpes zoster-related facial palsy (ZSH and RHS)

Year	Bell			ZSH		RHS			ZSH + RHS		Total APFP cases
	n	%		n	%	n	%		n	%	
2007	11	64.7		1	5.9	5	29.4		6	35.3	17
2008	30	69.8		7	16.3	6	14.0		13	30.2	43
2009	24	72.7		4	12.1	5	15.2		9	27.3	33
2010	30	65.2		5	10.9	11	23.9		16	34.8	46
2011	24	72.7		3	9.1	6	18.2		9	27.3	33
2012	34	63.0		10	18.5	10	18.5		20	37.0	54
2013	25	69.4		3	8.3	8	22.2		11	30.6	36
2014	24	57.1		11	26.2	7	16.7		18	42.9	42
2015	29	63.0		13	28.3	4	8.7		17	37.0	46
2016	34	70.8		8	16.7	6	12.5		14	29.2	48
2017	33	62.3		8	15.1	12	22.6		20	37.7	53
2018	25	59.5		7	16.7	10	23.8		17	40.5	42
2019	26	72.2		6	16.7	4	11.1		10	27.8	36
2020	18	52.9		6	17.6	10	29.4		16	47.1	34
2021	32	71.1		5	11.1	8	17.8		13	28.9	45
2022	23	69.7		6	18.2	4	12.1		10	30.3	33
Total	422	65.8		103	16.1	116	18.1		219	34.2	641

**Table 2:** Comparison of the proportion of Bell’s palsy and herpes zoster–related facial palsy before and after the introduction of routine varicella vaccination ZSH: zoster sine herpete; RHS: Ramsay Hunt syndrome Comparison of the proportion of patients with Bell’s palsy and HZ-related facial palsy (ZSH and RHS) among patients with acute peripheral facial paralysis between the prevaccination (2007-2014) and postvaccination (2015-2022) periods

Diseases	Before (2007-2014)			After (2015-2022)			Odds ratio	95% confidential interval	Chi-square value	p-value
	n	%		n	%					
Bell	202	66.4		220	65.3		0.949	0.685-1.316	0.097	0.803
HZ-related (ZSH and RHS)	102	33.6		117	34.7		1.053	0.760-1.460		

Age-specific rate of HZ in APFP patients

**Figure 2:**
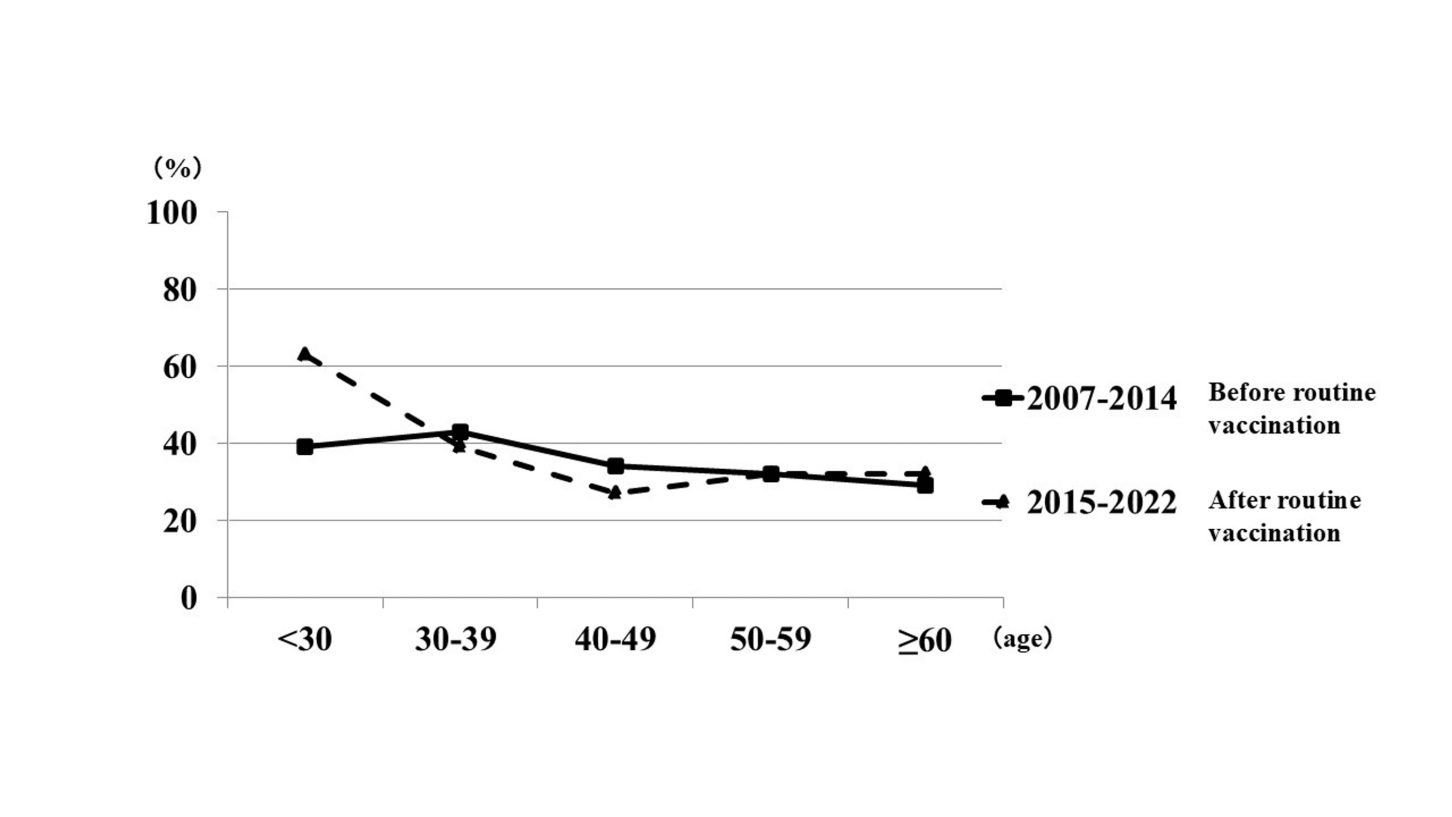
Age-specific change in the proportion of herpes zoster-related facial palsy before and after routine varicella vaccination ZSH: zoster sine herpete; RHS: Ramsay Hunt syndrome Line graphs comparing the age-specific proportions (%) of herpes zoster-related facial palsy (ZSH and RHS) among patients with acute peripheral facial paralysis during the prevaccination (2007-2014) and postvaccination (2015-2022) periods

Next, we analyzed the age-specific rates of HZ-related facial palsy during the prevaccination (2007-2014) and postvaccination (2015-2022) periods (Table 3). In patients aged 15-29 years, the proportion of HZ significantly increased from 38.9% (14/36) in the prevaccination period to 63.3% (19/30) in the post-vaccination period (odds ratio, 2.714; 95% CI, 1.009-7.301; χ² = 3.910; p = 0.048, chi-square test). This odds ratio indicates that patients in this age group had approximately 2.7 times higher odds of developing HZ-related facial palsy after the introduction of routine varicella vaccination compared with the prevaccination period, which is consistent with the statistically significant difference observed. In contrast, no significant differences were observed in any age group over 30 years between the two time periods. Figure 2 illustrates the changes in age-specific rates of HZ-related facial palsy across both periods.

**Table 3:** Age-specific proportion of herpes zoster-related facial palsy among patients with acute peripheral facial paralysis APFP: acute peripheral facial palsy; ZSH: zoster sine herpete; RHS: Ramsay Hunt syndrome Proportion of HZ-related facial palsy (ZSH and RHS) among patients with APFP by age group before and after the introduction of routine varicella vaccination. The table shows the number and percentage of HZ-related patients in each age category.

Age, yrs	Periods, yrs						Odds ratio	95% confidential interval	Chi-square value	p-value
	Before (2007-2014)			After (2015-2022)						
	ZHS and RHS (total APFP cases)	%		ZHS and RHS (total APFP cases)	%					
<30	14 (36)	38.9		19 (30)	63.3		2.714	1.009-7.301	3.910	0.048
30-39	18 (42)	42.9		16 (41)	39.0		0.853	0.358-2.034	0.130	0.723
40-49	17 (50)	34.0		16 (60)	26.7		0.706	0.314-1.587	0.698	0.403
50-59	18 (56)	32.1		21 (66)	31.8		0.985	0.462-2.100	0.001	0.970
≥60	35 (120)	29.2		45 (140)	32.1		0.869	0.513-1.473	0.264	0.604

## Discussion

In Japan, routine varicella vaccination was introduced in October 2014 for children aged 12-36 months old. Following its implementation, the number of reported varicella cases among children under five years of age declined rapidly [6]. As the incidence of varicella in children has decreased, the booster effect of natural infection has diminished. This raised concerns about a possible increase in HZ incidence among adolescents and a younger age at onset [9,10]. In the United States, the incidence of HZ among individuals aged 1-29 years has temporarily increased following the introduction of routine varicella vaccination [15]. However, more than 25 years after the program's initiation, the incidence has been declining [15].

The Miyazaki study, a large-scale epidemiological study in Japan [7], reported that the incidence of HZ was already increasing among older adults even prior to the introduction of routine vaccination in 2014. While the exact reasons remain unclear, contributing factors may include societal shifts toward nuclear families with fewer children, improved access to healthcare and increased consultation rates, and reduced natural boosting due to the milder clinical course of varicella and HZ resulting from widespread antiviral use. Furthermore, the Miyazaki study noted an increased incidence of HZ among child-rearing generations (20-40 years) following the introduction of routine vaccination [11], likely due to further attenuation of the natural booster effect.

In the present study, we investigated trends in the incidence of ZSH and RHS among patients with APFP in Hokkaido before and after the initiation of routine varicella vaccination. No significant change in the overall proportion of HZ-related facial palsy (ZSH and RHS) was observed. We observed annual fluctuations in the proportion of HZ cases, which may reflect known seasonal and year-to-year variability in VZV reactivation [7]. Notably, however, a significant increase in the proportion of HZ among patients aged 15-29 years was observed after the introduction of routine childhood varicella vaccination. This finding is consistent with the Miyazaki study, which reported a similar increase in this younger age group in Japan [11]. To provide a broader epidemiological context, we also referenced long-term data from the United States, where the incidence in this age group initially increased but subsequently declined after 25 years of widespread vaccination [15]. These international data are provided solely as epidemiological context and are not intended to imply any direct extrapolation from our findings.

The preceding section addressed age-specific trends in the incidence of HZ in relation to childhood varicella vaccination. In contrast, HZ prevention in older adults involves a different public-health strategy, namely, the use of zoster vaccines. The incidence and the severity of HZ tend to increase with age. Elderly patients also have a higher risk of developing postherpetic neuralgia, which significantly impairs their quality of life. A large US clinical trial demonstrated that HZ vaccination (Zostavax®) significantly reduced the incidence of both HZ and postherpetic neuralgia in individuals aged ≥50 years [16,17]. In Japan, the live attenuated varicella vaccine (BIKEN®) was approved in 2016 for HZ prevention in people aged 50 and older, as it contains a viral titer similar to that of Zostavax®. Additionally, the recombinant subunit vaccine Shingrix® was approved in 2018 [18]. With wider adoption of these vaccines, the incidence of ZSH and RHS may decrease.

Clinically, recovery rates for facial nerve palsy caused by ZSH and RHS remain modest, at approximately 60-70%, with many patients experiencing sequelae. While a meta-analysis of real-world data showed that Zostavax® reduced the incidence of ocular HZ by 30% [19], no such evidence currently exists for RHS. Moreover, HZ vaccines are currently approved only for individuals aged 50 and older, leaving younger patients ineligible. Therefore, careful diagnosis and appropriate treatment of APFP are especially important for patients aged 15-29 years.

This study had several limitations. First, we did not analyze the incidence of Bell’s palsy, ZSH, or RHS in the general population. Instead, we calculated only the proportion of ZHS and RHS among the APFP cases. This assumes that the overall incidence of Bell’s palsy remained stable throughout the study period. Unfortunately, no comprehensive epidemiological data on Bell’s palsy have been reported in Japan since a nationwide survey in collaboration with 28 medical facilities in 1988 [20]. However, in our cohort, the annual number of Bell’s palsy cases ranged from 23 to 34 (mean: 26.4), with no notable fluctuations. Second, this study was conducted at a single institution in northern Japan with a limited number of patients, making it difficult to determine whether the observed patterns are unique to our institution or reflect broader epidemiological trends. In addition, the diagnostic work-up and clinical diagnoses were performed at a single institution and may differ from practices at other centers, which could limit the generalizability and reproducibility of our findings. Nonetheless, our findings are consistent with those of a large-scale study conducted in Miyazaki Prefecture, southern Japan, particularly among younger patients [11]. Third, we should consider selection bias inherent to tertiary care hospital-based datasets, as well as potential misclassification bias between ZSH and Bell’s palsy. However, the use of standardized serological criteria to distinguish ZSH from Bell’s palsy represents a methodological strength of our study. The proportion of ZSH among patients diagnosed with Bell’s palsy (19.6%) was nearly identical to that reported in a Japanese multi-institutional study (18.7%) [2].

Although future studies incorporating multicenter or national registry data would substantially enhance the generalizability and relevance of the findings, our results provide a valuable baseline for future research on the incidence of HZ in APFP patients following the introduction of routine varicella vaccination in Japan. The rising incidence of HZ-related facial palsy among younger individuals in Hokkaido should be carefully considered in the diagnosis and management of APFP.

## Conclusions

In this 15-year cohort study of 641 patients with APFP in Hokkaido, Japan, no significant overall change was observed in the proportion of HZ-related cases, including ZSH and RHS, before and after the introduction of routine varicella vaccination in 2014. However, a significant increase in HZ among APFP patients aged 15-29 years suggests a reduction in VZV immunity due to decreased natural exposure following widespread childhood vaccination.

Although recovery rates for APFP caused by ZSH and RHS remain modest, early diagnosis and timely antiviral treatment are essential. Continued epidemiological surveillance and multicenter studies are warranted to validate these findings and to better understand the long-term impact of routine varicella vaccination on VZV reactivation in Japan.

## References

[1] Furuta Y, Fukuda S, Suzuki S, Takasu T, Inuyama Y, Nagashima K (1997). Detection of varicella-zoster virus DNA in patients with acute peripheral facial palsy by polymerase chain reaction and its use for early diagnosis of zoster sine herpete. J Med Virol.

[2] Kawaguchi K, Inamura H, Abe Y (2007). Reactivation of herpes simplex virus type 1 and varicella-zoster virus and therapeutic effects of combination therapy with prednisolone and valacyclovir in patients with Bell's palsy. Laryngoscope.

[3] Hato N, Yamada H, Kohno H (2007). Valacyclovir and prednisolone treatment for Bell's palsy: a multicenter, randomized, placebo-controlled study. Otol Neurotol.

[4] Robillard RB, Hilsinger RL Jr, Adour KK (1986). Ramsay Hunt facial paralysis: clinical analyses of 185 patients. Otolaryngol Head Neck Surg.

[5] Yoshikawa T, Kawamura Y, Ohashi M (2016). Universal varicella vaccine immunization in Japan. Vaccine.

[6] (2022). Japan Institute for Health Security. Changes in varicella outbreak trends after routine varicella vaccination-from the Survey of Infectious Disease Outbreak Trends, as of Week 26. https://id-info.jihs.go.jp/niid/ja/varicella-m/varicella-idwrs/10892-varicella-20220113.html.

[7] Toyama N, Shiraki K (2009). Epidemiology of herpes zoster and its relationship to varicella in Japan: a 10-year survey of 48,388 herpes zoster cases in Miyazaki prefecture. J Med Virol.

[8] Thomas SL, Wheeler JG, Hall AJ (2002). Contacts with varicella or with children and protection against herpes zoster in adults: a case-control study. Lancet.

[9] Garnett GP, Grenfell BT (1992). The epidemiology of varicella-zoster virus infections: the influence of varicella on the prevalence of herpes zoster. Epidemiol Infect.

[10] Papaloukas O, Giannouli G, Papaevangelou V (2014). Successes and challenges in varicella vaccine. Ther Adv Vaccines.

[11] Toyama N, Shiraki K (2018). Universal varicella vaccination increased the incidence of herpes zoster in the child-rearing generation as its short-term effect. J Dermatol Sci.

[12] Aizawa H, Ohtani F, Furuta Y, Sawa H, Fukuda S (2004). Variable patterns of varicella-zoster virus reactivation in Ramsay Hunt syndrome. J Med Virol.

[13] Aizawa H, Furuta Y, Ohtani F, Suzuki F, Fukuda S (2002). Fluctuations in anti-VZV IgG antibody for serological diagnosis of VZV reactivation in patients with acute peripheral facial palsy (Article in Japanese). Facial N Res Jpn.

[14] Kanda Y (2013). Investigation of the freely available easy-to-use software 'EZR' for medical statistics. Bone Marrow Transplant.

[15] Leung J, Dooling K, Marin M, Anderson TC, Harpaz R (2022). The impact of universal varicella vaccination on herpes zoster incidence in the United States: comparison of birth cohorts preceding and following varicella vaccination program launch. J Infect Dis.

[16] Oxman MN, Levin MJ, Johnson GR (2005). A vaccine to prevent herpes zoster and postherpetic neuralgia in older adults. N Engl J Med.

[17] Schmader KE, Levin MJ, Gnann JW Jr (2012). Efficacy, safety, and tolerability of herpes zoster vaccine in persons aged 50-59 years. Clin Infect Dis.

[18] Lal H, Cunningham AL, Godeaux O (2015). Efficacy of an adjuvanted herpes zoster subunit vaccine in older adults. N Engl J Med.

[19] Mbinta JF, Nguyen BP, Awuni PMA, Paynter J, Simpson CR (2022). Post-licensure zoster vaccine effectiveness against herpes zoster and postherpetic neuralgia in older adults: a systematic review and meta-analysis. Lancet Healthy Longev.

[20] Koike Y (1988). An epidemiological and clinical study on idiopathic facial palsy in Japan. Acta Otolaryngol Suppl.

